# Enhanced detection and characterization of germline structural variants in cancer predisposition genes via genome sequencing

**DOI:** 10.1016/j.gimo.2025.103459

**Published:** 2025-09-19

**Authors:** Parisa K. Kargaran, Qiliang Ding, Lauren A. Choate, Heidi L. Sellers, Mariam I. Stein, Belle A. Moyers, Shubham Basu, Pratyush P. Tandale, Rohit Setlem, Megan F. Bishop, Megan A. Holdren, Rhianna M. Urban, Sounak Gupta, Wei Shen

**Affiliations:** 1 Department of Cardiovascular Medicine, Center for Regenerative Medicine, Mayo Clinic, Rochester, MN; 2 Division of Laboratory Genetics and Genomics, Department of Laboratory Medicine and Pathology, Mayo Clinic, Rochester, MN; 3 Division of Computational Biology, Department of Quantitative Health Sciences, Mayo Clinic, Rochester, MN

**Keywords:** Cancer predisposition genes, Copy-number variant, Genome sequencing, Intragenic duplication, Structural variant

## Abstract

**Purpose:**

Germline pathogenic variants in cancer predisposition genes are found in approximately 10% of all cancer cases. Although multigene panel testing is the current first-tier approach for detecting variants in these genes, it has limitations in identifying and characterizing copy-number variants and other structural variants (SVs). Genome sequencing (GS) provides a more uniform coverage throughout the genome, thereby offering a more comprehensive method for copy-number variant and SV detection; however, its diagnostic utility in genetic testing for cancer predisposition remains underexplored.

**Methods:**

In this study, we performed GS on 33 patients with previously identified germline SVs in cancer predisposition genes, including 25 deletions, 7 duplications, and 1 mobile element insertion.

**Results:**

Using 2 SV callers in the DRAGEN pipeline, GS achieved 100% sensitivity in detecting these SVs. Moreover, GS revealed additional insights not available through previous clinical testing in 9 (27%) cases, including identifying a complex SV, clarifying structural configuration of intragenic duplications, and refining breakpoints at base-pair resolution.

**Conclusion:**

Taken together, our findings support the utility of GS as the sequencing backbone for germline genetic testing of cancer predisposition genes with improved detection, characterization, and clinical interpretation of SVs.

## Introduction

Approximately 10% of all cancer cases are associated with germline pathogenic variants in cancer predisposition genes.^1^ Germline genetic testing, most commonly through multigene panels (hereafter referred to as “panel testing”), has increasingly become the standard of care for patients newly diagnosed with cancer.^2^ These panels are most commonly performed using targeted next-generation sequencing (NGS), in which exons and their flanking regions are selectively amplified or captured and then sequenced. The clinical utility of panel testing for cancer predisposition genes is well established (eg, Whitworth et al^3^); however, its technical limitations may affect the detection and interpretation of copy-number variants (CNVs) and other structural variants (SVs). SVs are genomic rearrangements encompassing at least 50 nucleotides, which may involve alterations in copy number, orientation, and/or locations. SVs can be categorized into CNVs (deletions and duplications) and copy-neutral SVs (eg, inversions, translocations, mobile element insertions [MEI]).^4^^,^^5^

Previous studies have shown that SVs, particularly CNVs, are an important source of germline pathogenic variants in cancer predisposition genes.^6^^,^^7^ Deletions can eliminate a full gene, remove exons encoding important functional domains, disrupt reading frame, or affect regulatory elements (eg, promoters).^8^ For example, deletion of *BRCA1* (HGNC: 1100) exon 8^6^ and *CHEK2* (HGNC: 16627) exons 9-10^9^ have been associated with breast cancer predisposition (OMIM 604370, 609265), and *BRCA1* full-gene deletion has been described in 2 Spanish families with hereditary breast and ovarian cancer.^10^ The clinical interpretation of deletions is relatively straightforward because the partial or complete loss of a cancer predisposition gene typically results in loss of function and is therefore considered pathogenic.^11^ On the other hand, the effect of duplications, particularly intragenic ones, are more challenging to interpret.^7^ When an intragenic duplication occurs in tandem and in the direct orientation, it may disrupt the reading frame, leading to loss of function. However, if a duplication with the same genomic content is inserted elsewhere in the genome (insertional duplication) without disrupting another gene, it is more likely to be benign. Thus, accurate interpretation of intragenic duplications often requires characterization of their orientation and breakpoint locations.

SV detection in clinical settings has relied on various strategies, eg, chromosomal microarray analysis (CMA), multiplex ligation-dependent probe amplification (MLPA), targeted NGS (including panels and exome sequencing), and genome sequencing (GS).^12^ These techniques vary in their ability to detect SVs based on type and size.^13^^,^^14^ CMA and MLPA are effective at identifying CNVs but generally cannot detect copy-neutral SVs. In rare cases, MLPA probes can be designed to target known copy-neutral SVs (eg, the 10-Mb pathogenic inversion affecting *MSH2* (HGNC: 7325), detectable by the MRC-Holland probemix P003). MLPA, being a targeted approach, is limited to predefined regions and cannot identify CNVs outside of the targeted loci. In addition, neither CMA nor MLPA can determine the orientation or exact breakpoints of duplications. Targeted NGS may detect CNVs; however, because read depth is uneven across the targeted regions, its ability in detecting small CNVs (eg, single-exon events) is limited.^14^ It is also unable to detect copy-neutral SVs unless breakpoints occur within or adjacent to exonic regions. Additionally, similar to CMA and MLPA, targeted NGS generally cannot resolve the orientation or breakpoints of duplications. GS provides a relatively even coverage across the genome and thus offers comprehensive SV deletion capabilities. It can detect both CNVs and copy-neutral SVs (including MEIs).^15^^,^^16^ In particular, it can detect variants with breakpoints outside genomic regions targeted by gene panels or exome sequencing.^17^ In addition, based on read patterns near breakpoints, GS may help resolve structural configurations of duplications.

Detection of SVs from GS data relies on variant callers,^18^ which can be broadly categorized based on their underlying algorithms: read depth-based callers and anomalous reads-based callers. Read depth-based callers, eg, CNVnator,^19^ can only detect CNVs but not copy-neutral SVs. These callers detect CNVs by identifying changes in read depth (decreases for deletions and increases for duplications) within the CNV interval. Some read depth-based callers, such as CNVpytor,^20^ also utilize allelic imbalance information to improve CNV detection. Anomalous reads-based callers, eg, Manta,^21^ detect SVs by identifying unexpected reads (eg, split reads) and/or read pairs (for paired-end sequencing, eg, with unusual insert sizes or reads pair orientations) that are not expected in the reference genome but are consistent with the presence of a potential SV. These callers can detect both CNVs and copy-neutral SVs. Combination of multiple methods, eg, as implemented in DELLY2,^22^ may improve the sensitivity of SV detection.

A number of studies have evaluated the utility of GS in germline genetic testing of cancer predisposition syndromes.^23^, ^24^, ^25^, ^26^ For example, a recent Australian study identified pathogenic variants in 5.1% of 195 cancer-affected patients.^23^ However, most pathogenic variants detected in previous studies were short-nucleotide variants found in or adjacent to exons, variants that arguably could also be identified by expanded panel testing. As a result, a potential unique key strength of GS (ie, better identification and characterization of SVs) has yet to be fully assessed. On the other hand, the strength of GS in detecting SVs in somatic cancer settings has been demonstrated, eg, in a cohort of over 250 patients with myeloid neoplasm, GS identified all translocations and copy-number alterations detected by cytogenetic analysis, as well as additional clinically significant findings.^27^ In this study, we specifically evaluated the utility of GS for detecting germline SVs in cancer predisposition genes. By deploying 2 SV callers, one based on read depth and the other on anomalous reads, we revealed that GS has superior potential for detecting pathogenic SVs in cancer predisposition genes. In addition, GS provided additional characterizations, not available through prior clinical testing, into the configurations of SVs in 9 (27%) cases, thereby improving the interpretation of their pathogenicity. Overall, our findings support the use of GS as sequencing backbone for cancer predisposition syndromes testing.

## Materials and Methods

Our cohort contained 33 patients who underwent genetic testing for cancer predisposition syndromes at an academic medical center. This cohort was compiled based on the presence of SVs affecting cancer predisposition genes, as previously detected during clinical testing. Comparator techniques used for clinical testing included quantitative polymerase chain reaction (qPCR), Sanger sequencing, MLPA, CMA, and targeted NGS. Primer sequences for qPCR and Sanger sequencing are included in Supplemental Table 1. The MRC-Holland MLPA kits were used for *BRCA1* (P002, version D1), *FLCN* (HGNC: 27310; P256, version B2, B4, or C1), and *PMS2* (HGNC: 9122; P008, version C1). MLPA for *VHL* (HGNC: 12687) used an in-house developed probeset. CMA was performed using 2 platforms: the Thermo Fisher CytoScan HD array and the Agilent SurePrint G3 Custom comparative genomic hybridization (CGH) array. The resolution of the CytoScan HD array is generally 30 kb for deletions and 60 kb for duplications (with higher resolution in probe-dense regions). The Agilent array targets the following cancer predisposition genes: *APC* (HGNC: 583), *AXIN2* (HGNC: 904), *BMPR1A* (HGNC: 1076), *CDH1* (HGNC: 1748), *CHEK2*, *EPCAM* (HGNC: 11529), *MLH1* (HGNC: 7127), *MLH3* (HGNC: 7128), *MSH2*, *MSH6* (HGNC: 7329), *PTEN* (HGNC: 9588), *SCG5-GREM1* (HGNC: 10816, 2001), *SMAD4* (HGNC: 6770), *STK11* (HGNC: 11389), and *TP53* (HGNC: 11998), and reports deletions and duplications spanning at least 5 probes. For targeted NGS, data for cancer predisposition genes were sliced from exome sequencing based on the IDT xGen Exome chemistry. SVs were detected based on read depth and/or anomalous reads and confirmed by orthogonal methods before reporting.

GS was performed on DNA extracted from these patients, with library preparation using the Illumina DNA PCR-free Prep kit and sequenced on the NovaSeq 6000 instrument (Illumina).^28^ Bioinformatic analyses were performed using the Illumina DRAGEN pipeline (https://developer.illumina.com/dragen), version 3.8 or 4.2. Reads were aligned to the GRCh38/hg38 human reference genome. The DRAGEN pipeline contained 2 variant callers for SV detection: a read depth-based CNV caller (referred to as the “CNV caller” thereafter) and an anomalous reads-based SV caller developed from Manta (referred to as the “SV caller” thereafter).^21^ The Integrative Genomics Viewer (IGV) version 2.13.2 was used for manual review of the alignment files.^29^

## Results

The goal of this study is to evaluate the performance of GS in detecting CNVs and other SVs affecting cancer predisposition genes. We compiled a cohort of 33 patients with known SVs in cancer predisposition genes. These SVs were previously identified through clinical assays including qPCR, Sanger sequencing, fragment analysis, MLPA, CMA, and targeted NGS (Table 1, Supplemental Table 2). The participants had a mean age ± standard deviation of 40 ± 19 years, with 18 males (55%) and 15 females (45%). Our cohort contained 25 deletions, 7 duplications, and 1 MEI (Alu insertion). We performed paired-end GS on these samples, followed by bioinformatics analyses using the DRAGEN pipeline. Specifically, 2 callers in the DRAGEN pipeline were used for SV detection, one based on read depth (hereafter referred to as the “CNV caller”) and another based on anomalous reads (hereafter referred to as the “SV caller”).

**Table 1:** The 33 patients analyzed by this study

ID	Clinical Assay	Clinical Results	CNV Caller Results	SV Caller Results	SV Coordinates per GS^a^	Size	Clinical Classification^b^	GS Classification^c^	ACMG Classification Criteria	Clinical Presentation	In gnomAD^d^	In ClinVar^e^
**Deletion**												
1	Targeted NGS, qPCR	*MSH2* (NM_000251.3), exon 13 deletion	Deletion	Deletion	2:47,475,758–47,477,948	2.2 kb	Pathogenic	Pathogenic	2E (0.9 pts); 4E (0.1 pts)	Family history of Lynch syndrome (OMIM 120435)	No	417439
2	CMA (Agilent aCGH)	*MSH2* (NM_000251.3), exons 1–6 deletion	Deletion	Deletion	2:47,402,360–47,422,404	20.0 kb	Pathogenic	Pathogenic	2C-1 (0.9 pts); 4E (0.3 pts)	Person history of rectal cancer	1/126,092 alleles	90496
3	CMA (Agilent aCGH)	*EPCAM* (NM_002354.3), exons 8–9 deletion^f^	Deletion	Deletion	2:47,377,963–47,391,090	13.1 kb	Pathogenic	Pathogenic	2A (1.0 pts)	Family history of Lynch syndrome (OMIM 613244)	No	417532
4	Targeted NGS, qPCR	*MLH1* (NM_000249.3), exon 15 deletion	No Call	Deletion	3:37,041,567–37,042,959	1.4 kb	Pathogenic	Pathogenic	2E (0.9 pts); 4E (0.1 pts)	Family history of Lynch syndrome (OMIM 609310)	No	No
5	CMA (Agilent aCGH)	*MLH1* (NM_000249.3), exon 13 deletion	Deletion	Deletion	3:37,028,354–37,030,696	2.3 kb	Pathogenic	Pathogenic	2E (0.9 pts); 4E (0.1 pts)	Family history of Lynch syndrome	No	417350
6	MLPA	*VHL* (NM_000551.4), exon 2 deletion	Deletion	Deletion	3:10,142,571–10,148,541	6.0 kb	Pathogenic	Pathogenic	2E (0.9 pts); 4E (0.3 pts)	Family history of von Hippel-Lindau disease (OMIM 193300)	No	660147
7	MLPA	*VHL* (NM_000551.4), exon 3 deletion	Deletion	Deletion	3:10,148,029–10,151,993	4.0 kb	Pathogenic	Pathogenic	2D-2 (0.9 pts); 4E (0.3 pts)	Family history of von Hippel-Lindau disease	No	651806
8	CMA (Agilent aCGH)	*APC* (NM_000038.5), exon 5 deletion	Deletion	Deletion	5:112,774,175–112,778,068	3.9 kb	Pathogenic	Pathogenic	2E (0.9 pts); 4E (0.3 pts)	Familial *APC* pathogenic variant	No	217980
9	MLPA, fragment analysis^g^	*PMS2* (NM_000535.7), exon 12 deletion	No Call	Deletion	7:5,982,373–5,983,278	906 bp	Pathogenic	Pathogenic	2E (0.9 pts); 5H (0.3 pts)	Family history of Lynch syndrome (OMIM 614337); mother also carrier of this deletion, had endometrial cancer with loss of PMS2 staining	No	No
10	MLPA	*PMS2* (NM_000535.7), exons 5–9 deletion	Deletion	Deletion	7:5,991,238–6,003,487	12.3 kb	Pathogenic	Pathogenic	2E (0.9 pts); 4E (0.1 pts)	Family history of Lynch syndrome	No	417545, 583745
11	CMA (CytoScan HD)	*PTEN* (NM_000314.8), exon 7 deletion	Deletion	Deletion	10:87,954,849–87,958,465	3.6 kb	Pathogenic	Pathogenic	2E (0.9 pts); 4E (0.1 pts)	Autism, developmental disorder	No	No
12	CMA (Agilent aCGH)	*PTEN* (NM_000314.8), exon 9 deletion	Deletion	Deletion	10:87,964,681–87,968,186	3.5 kb	Pathogenic	Pathogenic	2D-2 (0.9 pts); 4E (0.1 pts)	Familial *PTEN* pathogenic variant	No	No
13	Targeted NGS, Sanger sequencing	*BRCA2* (NM_000059.3), c.8975_9100del, p.Pro2992_Thr3033del	No Call	Deletion	13:32,379,771–32,379,896	126 bp	Likely Pathogenic	Likely Pathogenic	PS4_Moderate, PM2, PM4	Not available	No	96876
14	MLPA	*BRCA1* (NM_007294.4), exons 1–2 deletion	Deletion	No Call	17:43,121,517–43,156,521	35.0 kb (approx.)	Pathogenic	Pathogenic	2C-1 (0.9 pts); 5G (0.1 pts)	Family history of breast cancer	No	584147
15	qPCR	*BRCA1* (NM_007294.4), exon 2 deletion	No Call	Deletion	17:43,123,996–43,124,962	967 bp	Pathogenic	Pathogenic	2E (0.9 pts); 5G (0.1 pts)	Family history of ovarian cancer	No	254080
16	qPCR	*BRCA1* (NM_007294.4), exons 13–15 deletion	Deletion	Deletion	17:43,068,061–43,078,984	10.9 kb	Likely Pathogenic	Likely Pathogenic	2E (0.9 pts)	Not available	No	No
17	MLPA	*BRCA1* (NM_007294.4), exon 19 deletion	Deletion	Deletion	17:43,053,392–43,057,564	4.2 kb	Pathogenic	Pathogenic	2E (0.9 pts); 5G (0.1 pts)	Family history of breast and ovarian cancer	No	462183, 583620
18	MLPA	*BRCA1* (NM_007294.4), exon 16 deletion	Deletion	Deletion	17:43,067,524–43,070,202	2.7 kb	Pathogenic	Pathogenic	2E (0.9 pts); 5G (0.1 pts)	Family history of breast cancer	No	89067
19	Sanger sequencing	*TP53* (NM_000546.6), c.283_375+21del114, p. Ser95_Thr125del	No Call	Deletion	17:7,675,973–7,676,086	114 bp	Likely Pathogenic	Likely Pathogenic	PVS1, PM2	Personal history of melanoma, thyroid cancer, brain cancer, family history of sarcoma and brain cancer	No	406569
20	CMA (CytoScan HD)	Suggestive of *TP53* promoter deletion^h^	Deletion	Deletion	17:7,685,488–7,689,988	4.5 kb	Inconclusive	Pathogenic	2C-2 (0.45 pts); 4E (0.3 pts); 4G (0.3 pts); 5G (0.1 pts)	Personal history of osteosarcoma in childhood	No	254052, 652964
21	MLPA	*FLCN* (NM_144997.7), exons 6–8 deletion	Deletion	Deletion	17:17,221,294–17,224,506	3.2 kb	Pathogenic	Pathogenic	2E (0.9 pts); 5G (0.1 pts)	Clinical diagnosis of Birt-Hogg-Dubé syndrome (OMIM 135150)	No	No
22	MLPA	*FLCN* (NM_144997.7), exon 1 deletion	Deletion	Deletion	17:17,236,469–17,243,207	6.7 kb	Pathogenic	Pathogenic	2C-2 (0.45 pts); 4E (0.3 pts); 5H (0.3 pts)	Personal history of pneumothorax	No	No
23	CMA (Agilent aCGH)	*SMAD4* (NM_005359.6), exons 2–12 deletion	Deletion	Deletion	18:51,034,475–51,472,263	437.8 kb	Pathogenic	Pathogenic	2D-4 (0.9 pts); 5G (0.1 pts)	Personal history of juvenile polyposis	No	No
24	CMA (Agilent aCGH)	*STK11* (NM_000455.5), exon 1 deletion	Deletion	Deletion	19:1,204,244–1,213,268	9.0 kb	Pathogenic	Pathogenic	2C-1 (0.9 pts); 4E (0.3 pts)	Possible diagnosis of Peutz-Jeghers syndrome (OMIM 175200)	No	No
25	CMA (Agilent aCGH), qPCR	*STK11* (NM_000455.5), exons 2–5 deletion	Deletion	Deletion	19:1,215,827–1,221,236	5.4 kb	Pathogenic	Pathogenic	2E (0.9 pts); 4E (0.1 pts)	Personal history of colon polyps	No	527861
Duplication												
26	CMA (Agilent aCGH)	*MLH1* (NM_000249.3), exons 16–19 duplication	Duplication	Tandem Duplication	3:37,045,688–37,052,536	6.8 kb	VUS	Likely Benign^i^	2J (0 pts)	Family history of colon cancer	5/126,092 alleles	237319, 584368 (VUS)
27	CMA (Agilent aCGH, CytoScan HD)	*APC* (NM_000038.5), exons 2–5 duplication	Duplication	Deletion	5:112,749,775–112,779,219	29.4 kb (approx.)	VUS	VUS^j^	2I (0 pts)	Incidentally detected on prenatal microarray; no known family history of cancer	No	469689, 657378 (VUS)
28	CMA (Agilent aCGH, CytoScan HD)	*SCG5-GREM1* locus, *SCG5* (NM_001144757.3), exon 6 duplication	Duplication	Tandem Duplication	15:32,695,006–32,718,858	23.9 kb	Pathogenic	Pathogenic	2A (1.0 pts)	Personal history of colorectal adenoma	No	No
29	MLPA	*BRCA1* (NM_007294.4), exon 12 duplication	Duplication	Tandem Duplication	17:43,078,282–43,084,362	6.1 kb	Pathogenic	Pathogenic	2I (0.9 pts); 5G (0.1 pts)	Family history of breast cancer	3/126,092 alleles	188222, 583774
30	Targeted NGS	*BRCA1* (NM_007294.4), exon 12 duplication	Duplication	Tandem Duplication	17:43,078,282–43,084,362	6.1 kb	Pathogenic	Pathogenic	2I (0.9 pts); 5G (0.1 pts)	Personal history of ovarian cancer	3/126,092 alleles	188222, 583774
31	MLPA	*BRCA1* (NM_007294.4), exon 16 duplication	Duplication	Tandem Duplication	17:43,066,858–43,069,973	3.1 kb	VUS	Pathogenic	2I (0.9 pts); 5G (0.1 pts)	Family history of breast cancer	No	645354
32	MLPA	*BRCA1* (NM_007294.4), exon 16 duplication	Duplication	Tandem Duplication	17:43,066,858–43,069,973	3.1 kb	VUS	Pathogenic	2I (0.9 pts); 5G (0.1 pts)	Family history of breast and ovarian cancer	No	645354
Mobile element insertion												
33	Targeted NGS	*BRCA2* (NM_000059.3), c.156_157insAlu	No Call	Insertion	13:32,319,151	N/A	Pathogenic	Pathogenic	Not applicable	Not available	No	126018

See Supplemental Table 2 for HGVS-compliant nomenclature for the variants.

*aCGH*, array comparative genomic hybridization; *ACMG,* American College of Medical Genetics and Genomics*;approx.*, approximate; *CNV*, copy-number variant; *GS*, genome sequencing; *PCR*, polymerase chain reaction; *pts*, points; *SV*, structural variant; *VUS*, variant of uncertain significance.

a If the SV caller detected the variant, the coordinates are from the SV caller because it has precise breakpoint information. Otherwise, the coordinates are from the CNV caller.

b Classification based on the clinical testing results.

c Classification based on the GS results.

d Based on gnomAD v4.1.0 SV.

e Including variants with identical exon content. If present, the ClinVar variation IDs are provided. Unless otherwise specified, the variants were classified as pathogenic or likely pathogenic by the ClinVar submitters.

f GS revealed that this deletion spanned exons 6-9 of *EPCAM*.

g Long-range PCR, followed by fragment analysis, confirmed that the deletion was in *PMS2*, not in the pseudogene *PMS2CL*.

h CMA revealed decreased signal for several probes upstream of *TP53*; however, the number of probes impacted was below the validated threshold for this microarray platform.

i GS revealed that this was a partial gene duplication that did not disrupt *MLH1* (instead of an intragenic duplication). Although this variant would have been classified as a VUS if strictly following the ACMG/ClinGen guidelines, laboratory director discretion was applied to classify it as likely benign (because it did not disrupt *MLH1*).

j GS revealed that this was a complex SV.

**Figure 1:**
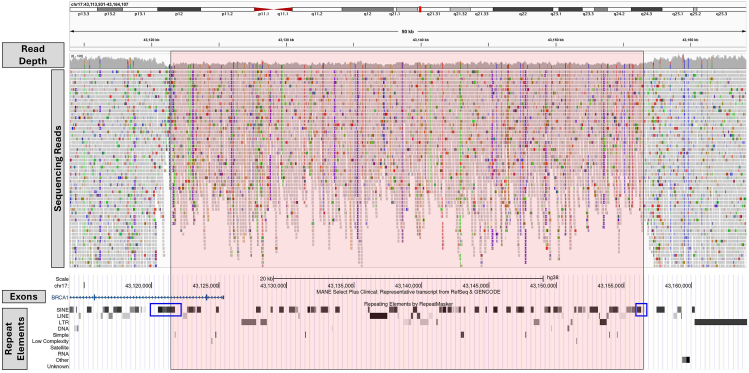
An approximately 35-kb pathogenic deletion affecting exons 1-2 of *BRCA1* that was detected by the CNV caller but missed by the SV caller in case 14. The red shaded region denotes the deletion interval identified by the CNV caller. Both breakpoints were in low-mappability SINE elements (blue boxes). CNV, copy-number variant.

Our cohort included 25 deletions, ranging in size from 114 bp to 437.8 kb. All deletions were detected by at least 1 of the 2 callers. Specifically, 19 deletions were identified by both callers, 5 were only detected by the SV caller, and 1 was only detected by the CNV caller. The 5 deletions missed by the CNV caller were generally small (114 bp-1.4 kb), which is consistent with DRAGEN’s guidance that the resolution of the CNV caller is approximately 1 kb for GS. This observation suggests that the anomalous reads-based SV caller has higher sensitivity for detecting smaller deletions. Conversely, the SV caller failed to detect a pathogenic ∼35-kb deletion affecting exons 1 and 2 of *BRCA1* (NM_007294.4). This deletion was successfully detected by the CNV caller, with approximate breakpoints at 17:43,121,517-43,156,521 (GRCh38/hg38). For this deletion, both breakpoints were located within short interspersed nuclear elements (SINE; Alu and MIR, respectively), which are not uniquely mappable (Figure 1). We suspect this is why the SV caller failed to identify the deletion: the SV caller relied on anomalous reads, including split reads, and if one or both breakpoints are located within low-mappability regions (eg, transposable elements or low-copy repeats), they may be missed by the SV caller. These findings highlight an important limitation of the SV caller and suggest that using both CNV and SV callers improves sensitivity of detecting deletions.

**Figure 2:**
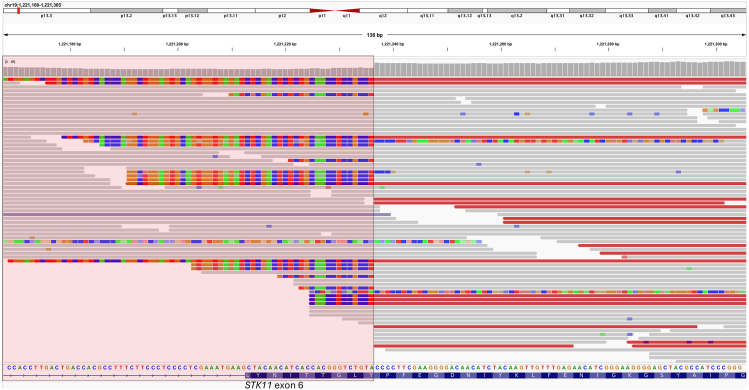
**A 5.4-kb likely pathogenic deletion affecting exons 2-6 of *STK11* in case 25.** In this patient, previous clinical testing (CMA and qPCR) revealed deletion of *STK11* exons 2-5. As shown in this figure, GS further clarified that the deletion had extended into exon 6, removing the first 24 nucleotides of this exon. CMA, chromosomal microarray analysis; GS, genome sequencing; qPCR, quantitative polymerase chain reaction.

For 2 of the 25 deletions, GS provided additional insights that were not available through prior clinical testing. A patient in his 30s (case 25), referred for hereditary colon cancer testing due to polyps, initially underwent CMA followed by qPCR confirmation, which identified a pathogenic deletion of *STK11* (NM_000455.5) exons 2-5. GS revealed that this deletion not only removed exons 2-5 but also extended into exon 6, partially deleting it (Figure 2). The qPCR probe for exon 6 was located outside the deleted region (Supplemental Table 1), explaining why the partial deletion of exon 6 was not detected by qPCR. Although this finding is unlikely to alter the variant classification in this case, precise identification of deletion breakpoints may be crucial in other cases because it is required for predicting reading frame disruptions and overall pathogenicity. Another patient in her 30s (case 20) underwent CMA testing because of suspected Li-Fraumeni syndrome (OMIM 151623). CMA detected decreased signal of several probes upstream of *TP53*. However, the number of affected probes was below the validated threshold of this microarray platform; therefore, the results were reported as inconclusive. Using GS, we confirmed that this deletion extended into exon 1 of *TP53*, which allowed for a pathogenic classification.

All 7 duplications, ranging from 3.1 to 29.4 kb in size, were detected by at least 1 of the 2 callers. In 6 cases, both the CNV and SV callers detected the duplications. In these cases, the SV caller confirmed that the duplications were in tandem, a crucial piece of information not available through prior clinical testing (eg, MLPA). This additional insight led to the reclassification of variant pathogenicity in 3 cases. Two of the reclassified cases involved a *BRCA1* exon 16 duplication (NM_007294.4; cases 31 and 32). Using MLPA, both patients were found to carry the duplication, which was initially interpreted as a variant of uncertain significance (VUS) because its configuration could not be determined. With GS, the duplications were proven to be in tandem in both patients, allowing for a pathogenic classification. In the third case, CMA detected a duplication of *MLH1* exons 16-19 (NM_000249.3; case 26), which was initially interpreted as a VUS because of the inability to resolve whether it was an intragenic or partial gene duplication. GS confirmed it as a partial gene duplication that extended beyond the 3′-end of *MLH1*, with *MLH1* remaining intact; thus, this variant was reclassified as likely benign.

**Figure 3:**
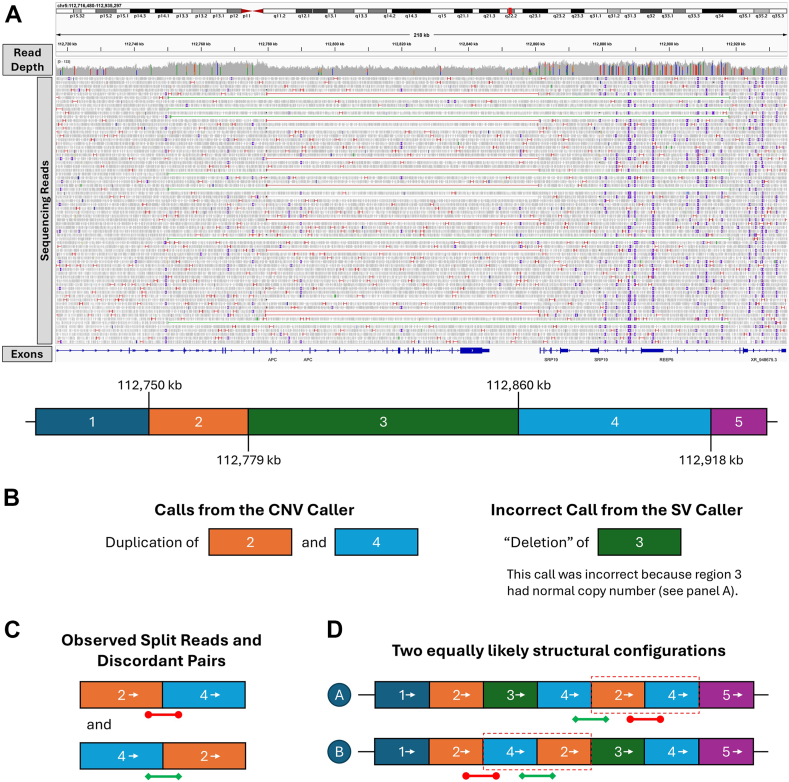
**The complex SV involving *APC* in case 27.** A. Regions 2-4 in the reference genome were involved in the complex SV. Region 2 contains exons 2-5 of *APC.* Region 3 contains exon 6 to the 3′-end of *APC*. Region 4 contains *SRP19* (HGNC: 11300) and *REEP5* (HGNC: 30077), 2 genes with no known disease associations. B. The CNV caller correctly identified duplications of regions 2 and 4, whereas the SV caller incorrectly identified a deletion involving region 3. C. Upon inspection of the alignment file, 2 groups of split reads and discordant read pairs connecting regions 2 and 4 were found (red and green, correspond to the read pairs of the same color in [A]). D. Based on the copy-number profile and the anomalous reads, 2 equally likely structural configurations of this SV can be inferred, both of which involved “coduplication” of regions 2 and 4 (red dashed box). The reading frame of *APC* is disrupted in configuration B but not in configuration A; thus, this complex SV remained as a VUS. CNV, copy-number variant; SV, structural variant; VUS, variant of uncertain significance.

In the seventh case (case 27), the CNV caller detected a duplication of exons 2-5 of *APC* (NM_000038.6), with approximate coordinates 5:112,749,775-112,779,219 (GRCh38/hg38). This duplication was first detected incidentally by prenatal microarray in this patient’s fetus and was subsequently confirmed to be inherited from the patient. There is no known family history of cancer. The SV caller missed this duplication but instead identified a deletion involving an adjacent region (5:112,779,705-112,860,879). This deletion was not supported by read depths and therefore was initially dismissed as a false-positive variant call. Interestingly, careful inspection of the alignment file revealed that this patient harbored a complex SV involving the “coduplication” of 2 discrete genomic regions, separated by a segment of normal copy number (Figure 3). The coduplication cassette encompassed *APC* exons 2-5 (5:112,749,775-112,779,219), fused with an approximately 58-kb region located 81 kb downstream of the duplicated *APC* exons (5:112,859,900-112,918,222), in the same orientation. Because of the limitations of Illumina short-read sequencing, we were not able to definitively resolve the structure of this SV, with 2 possible configurations (Figure 3). Because *APC* remains intact in 1 of the 2 possible configurations, the variant remained classified as a VUS. Future investigations, eg, RNA studies, long-read sequencing, or optical genome mapping,^30^ are required to definitively resolve the pathogenicity of this variant.

**Figure 4:**
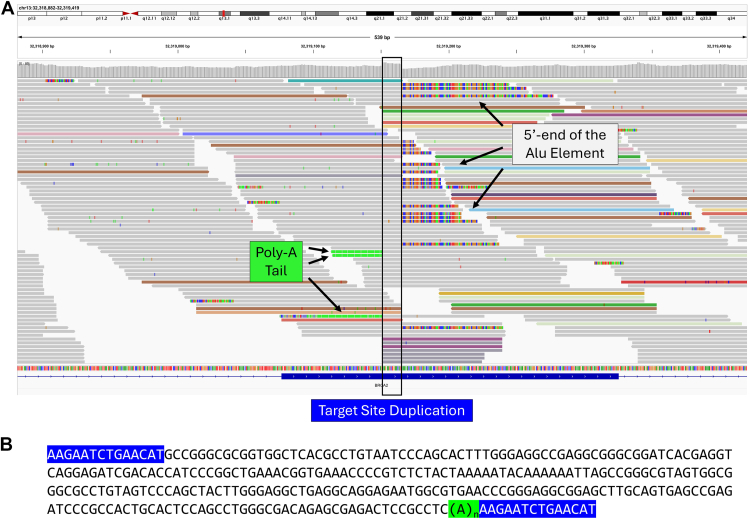
**An Alu insertion into *BRCA2* (HGNC: 1101) identified in case 33.** A. Manual review of the alignment file in IGV revealed hallmark features of an Alu insertion: a target site duplication, presence of the 5′-end sequence of an Alu element, and a poly-A tail. B. Paired-end reads were assembled to reconstruct the inserted sequence. BLAST analysis revealed highest similarity to the Ya5 subfamily of Alu elements. BLAST, Basic Local Alignment Search Tool; IGV, Integrative Genomics Viewer.

We also sequenced 1 sample with a pathogenic MEI in *BRCA2* (NM_000059.3):c.156_157insAlu. This MEI was successfully detected by the SV caller as expected. Manual review of the alignment file confirmed the presence of a poly-A tail and a 14-bp target site duplication sequence (Figure 4A), similar to previously described.^31^ Based on paired-end reads, we successfully assembled the full length of the Alu element, except for the poly-A tail (Figure 4B).

## Discussion

In this study, we demonstrated that GS, combined with 2 variant callers in the DRAGEN pipeline, successfully detected all known SVs involving cancer predisposition genes in all 33 patients, achieving 100% sensitivity. The CNV and SV callers showed complementary strengths: the SV caller had higher sensitivity for smaller CNVs (<1 kb) and successfully detected the pathogenic Alu insertion, whereas the CNV caller identified a deletion with breakpoints in low-mappability regions that was missed by the SV caller. This finding suggests that using both read depth-based and anomalous reads-based callers is crucial for comprehensive SV detection in clinical GS.

The complex SV in our cohort highlights the need of further investigation when CNV and SV callers provide conflicting results rather than immediately dismissing one result as a false positive. The anomalous reads-based SV caller, which operates under the assumption of simple SVs, may misclassify complex structural rearrangements, eg, calling a deletion when interspersed duplications (ie, “coduplication”) are present. In other words, despite potentially misclassifying the event, a variant call by the SV caller still serves as a useful indicator that an atypical structural rearrangement may be present. The complex *APC* duplication (Figure 3), for which we were unable to fully determine the structural configuration using short reads alone, also underscores the limitations of short-read sequencing in resolving complex SVs.

Our findings demonstrate that GS has superior capabilities in detecting and characterizing SVs affecting cancer predisposition genes, compared with targeted NGS panels and traditional molecular genetic methods. In our cohort of 33 patients harboring SVs affecting cancer predisposition genes, GS not only successfully uncovered all previously detected SVs but also provided additional insights critical for variant interpretation in 9 (27%) patients. In a patient with a previously identified *STK11* intragenic deletion (exons 2-5), GS revealed that the deletion extended into exon 6. Partial exon deletions are challenging to detect by qPCR, MLPA, or CMA because of probe placement; however, accurate breakpoint identification is required for predicting reading frame impact and, consequently, variant pathogenicity. In another patient, GS clarified an inconclusive CMA result and confirmed the presence of a pathogenic partial gene deletion involving *TP53*. In addition, among the 7 patients with duplications involving cancer predisposition genes, GS revealed that 6 were tandem duplications, a crucial piece of information that could not be determined through other clinical assays, eg, MLPA or panel testing. In the last case, GS revealed that the duplication was part of a complex SV involving *APC*, which supported maintaining its classification as a VUS. Interestingly, several complex SVs affecting *APC* have been documented in the literature.^32^^,^^33^ These results suggest that GS provides significant benefit for patients with SVs affecting cancer predisposition genes, eg, those with intragenic or partial gene duplications. Although the current guidelines^34^ allow us to presume duplications are in tandem, emerging evidence,^35^ including our case with *APC* exons 2-5 duplication, indicates that nontandem duplications may be more common than previously thought.

A limitation of our study is that the vast majority of SVs included in our study (32 of 33) were CNVs. This reflects the ascertainment methods used in clinical testing and the relative rarity of pathogenic copy-neutral SVs in cancer predisposition genes. Therefore, copy-neutral SVs, such as inversions and balanced translocations are underrepresented in our cohort. Although GS is, in principle, capable of detecting such rearrangements, we did not have access to clinically confirmed samples with such variants for inclusion. Future studies are warranted to fully assess the capabilities and limitations of GS in detecting these variants.

A key question remains whether GS should replace targeted NGS panels as the first-tier diagnostic tool for hereditary cancer predisposition syndromes. A recent Australian health economics analysis found that national adoption of diagnostic GS for cancer predisposition syndromes would necessitate a 9-fold increase in annual health care expenditure,^23^ thereby limiting its near-term feasibility as a first-tier test. A more practical alternative may be to use GS as the sequencing backbone, in conjunction with targeted gene panel reporting. In other words, GS would be used as the sequencing chemistry, with bioinformatics filters applied to selectively interpret only the variants affecting cancer predisposition genes. This is conceptually similar to the “capture backbone” (see SoRelle et al^36^). This approach balances cost-effectiveness with the improved SV detection and characterization while minimizing the variant review burden from untargeted GS and the associated cost. We are hopeful that clinical laboratories will increasingly adopt GS as their sequencing backbone, thereby benefiting patients with SVs affecting cancer predisposition genes.

## Data Availability

The data generated by this study are available from the corresponding authors upon request.

## Conflict of Interest

The authors declare no conflicts of interest.
