# Supplementary material for: Enhanced detection and characterization of germline structural variants in cancer predisposition genes via genome sequencing
Source: Genet Med Open. 2025 Sep 19;3:103459. doi: 10.1016/j.gimo.2025.103459 (PMC12590437; doi:10.1016/j.gimo.2025.103459)
Supplement: Supplemental Tables 1 and 2 [file mmc1.pdf]

Supplemental Materials

Enhanced Detection and Characterization of Germline Structural Variants in Cancer Predisposition Genes via Genome Sequencing

Supplemental Table 1. Primer sequences for qPCR and Sanger sequencing.

[1] The amplicon (19:1,221,258–1,221,340) is outside the deletion identified in Case 25 (19:1,215,827–1,221,236), explaining why the partial exon 6 deletion was not detected by qPCR.

Gene (exon)	Primer sequence
BRCA1 (exon 2)	Forward: 5'-CACTCTTGTGCTGACTTACCAG-3'<br>Reverse: 5'-GCTCTTCGCGTTGAAGAAGTA-3'
BRCA1 (exon 13)	Forward: 5'-CCTTTCCACTCCTGGTTCTTTA-3'<br>Reverse: 5'-TGAATACCCTATAAGCCAGAATCC-3'
BRCA1 (exon 15)	Forward: 5'-TTTGTTGACCCTTTCTGTTGAAG-3'<br>Reverse: 5'-ACTACTGATACTGCTGGGTATAATG-3'
BRCA2 (exon 23)	Forward: 5'-TGGCGTCCATCATCAGATTTAT-3'<br>Reverse: 5'-TTGTCGCTGCTAACTGTATGT-3'
MLH1 (exon 15)	Forward: 5'-GTGAAGAACTGTTCTACCAGAT-3'<br>Reverse: 5'-AGGATCTAAACTTACCGATAACC-3'
MSH2 (exon 13)	**Primer set 1**<br>Forward: 5'-CCCAATATGGGAGGTAAATCAACA-3'<br>Reverse: 5'-CACTTCTGCTGACTCACATGG-3'<br>**Primer set 2**<br>Forward: 5'-TTGTGGACTGCATCTTAGCC-3'<br>Reverse: 5'-AGGGACTAGGAGATGCACTTA-3'
STK11 (exon 2)	Forward: 5'-CAGGGAAATTCAACTACTGAGG-3'<br>Reverse: 5'-TTTCTGCTTCTCTTCGTTGT-3'
STK11 (exon 3)	Forward: 5'-CCCTGAGCTGTGTGTCCTTA-3'<br>Reverse: 5'-CTGTCCAGCATTTCCTGCAT-3'
STK11 (exon 4)	Forward: 5'-CTGATTGACGGCCTGGAGTA-3'<br>Reverse: 5'-CCTAGCACGTGCCTACCTC-3'
STK11 (exon 5)	Forward: 5'-CAGGCACTGCACCCGTT-3'<br>Reverse: 5'-CCAGGCCGTTGGCAATCT-3'
STK11 (exon 6)[1]	Forward: 5'-CTACAAGTTGTTTGAGAACATCG-3'<br>Reverse: 5'-CTTTCAGCAGGTCAGAGAGC-3'
TP53 (exon 4)	Forward: 5'-ATATTCAACTTTGGGACAGGAGT-3'<br>Reverse: 5'-GCAATGGATGATTTGATGCTGT-3'

Supplemental Table 2. HGVS-compliant nomenclature for the variants described in this study.

[1] Note that the coordinates in this column may differ from those in Table 1 due to the HGVS 3'-rule. [2] GS clarified that this nomenclature is inaccurate, as the duplication was found not in tandem. [3] The structural configuration of this variant has not been definitively resolved.

ID	Clinical Results	GS Results[1]
1	NC_000002.12:g.(47475271_47476366)_(47476572_47478271)del	NC_000002.12:g.47475760_47477950del
2	NC_000002.12:g.(?_47403191)_(47416430_47429741)del	NC_000002.12:g.47402370_47422414del
3	NC_000002.12:g.(?_47385165)_(47386614_?)del	NC_000002.12:g.47377978_47391105del
4	NC_000003.12:g.(37040295_37042267)_(37042332_37047518)del	NC_000003.12:g.37041569_37042961del
5	NC_000003.12:g.(37026008_37028783)_(37028933_37040185)del	NC_000003.12:g.37028354_37030696del
6	NC_000003.12:g.(10142188_10146513)_(10146637_10149786)del	NC_000003.12:g.10142571_10148541del
7	NC_000003.12:g.(10146637_10149786)_(10149966_?)del	NC_000003.12:g.10148049_10152013del
8	NC_000005.10:g.(112767391_112775628)_(112775738_112780789)del	NC_000005.10:g.112774180_112778073del
9	NC_000007.14:g.(5978697_5982823)_(5982992_5986758)del	NC_000007.14:g.5982396_5983301del
10	NC_000007.14:g.(5989956_5991972)_(6002637_6003689)del	NC_000007.14:g.5991242_6003491del
11	NC_000010.11:g.(87952260_87957852)_(87958020_87960893)del	NC_000010.11:g.87954849_87958465del
12	NC_000010.11:g.(87961119_87965286)_(87965473_?)del	NC_000010.11:g.87964683_87968188del
13	NM_000059.3:c.8975_9100del	NC_000013.11:g.32379771_32379896del
14	NC_000017.11:g.(43115780_43124016)_(43125365_?)del	NC_000017.11:g.43121517_43156521del
15	NC_000017.11:g.(43115780_43124016)_(43124116_43125270)del	NC_000017.11:g.43123996_43124962del
16	NC_000017.11:g.(43067696_43070927)_(43076615_43082403)del	NC_000017.11:g.43068071_43078994del
17	NC_000017.11:g.(43051118_43057051)_(43057136_43063332)del	NC_000017.11:g.43053415_43057587del
18	NC_000017.11:g.(43063952_43067607)_(43067696_43070927)del	NC_000017.11:g.43067534_43070212del
19	NM_000546.6:c.283_375+21del	NC_000017.11:g.7675973_7676086del
20	Inconclusive	NC_000017.11:g.7685502_7690002del
21	NC_000017.11:g.(17219210_17221536)_(17224144_17226175)del	NC_000017.11:g.17221294_17224506del
22	NC_000017.11:g.(17232902_17236911)_(17237169_?)del	NC_000017.11:g.17236469_17243207del
23	NC_000018.10:g.(51030624_51046919)_(51078468_?)del	NC_000018.10:g.51034478_51472266del
24	NC_000019.10:g.(?_1206913)_(1207204_1218416)del	NC_000019.10:g.1204268_1213292del
25	NC_000019.10:g.(1207204_1218416)_(1220718_1221212)del	NC_000019.10:g.1215830_1221239del
26	NC_000003.12:g.(37042332_37047518)_(37050654_?)dup	NC_000003.12:g.37045691_37052539dup
27	NC_000005.10:g.(112737926_112754872)_(112775738_112780789)dup[2]	Not available[3]
28	NC_000015.10:g.(32691764_32696513)_(32696610_?)dup	NC_000015.10:g.32695006_32718858dup
29	NC_000017.11:g.(43076615_43082403)_(43082576_43090943)dup	NC_000017.11:g.43078305_43084385dup
30	NC_000017.11:g.(43076615_43082403)_(43082576_43090943)dup	NC_000017.11:g.43078305_43084385dup
31	NC_000017.11:g.(43063952_43067607)_(43067696_43070927)dup	NC_000017.11:g.43066881_43069996dup
32	NC_000017.11:g.(43063952_43067607)_(43067696_43070927)dup	NC_000017.11:g.43066881_43069996dup
33	NM_000059.3:c.156_157insN[?]	NC_000013.11:g.32319165_32319166insN[?]
